# Supplementary material for: Nasendoscopy to Predict Difficult Videolaryngoscopy: A Multivariable Model Development Study
Source: J Clin Med. 2023 May 12;12(10):3433. doi: 10.3390/jcm12103433 (PMC10219304; doi:10.3390/jcm12103433)
Supplement: Supplementary file 1 [file jcm-12-03433-s001.zip › jcm-2376539-supplementary.pdf]

ELECTRONIC SUPPLEMENTARY MATERIAL

Sasu P.B. *et al.*: Nasendoscopy to predict difficult videolaryngoscopy: a multivariable model development study

Disclaimer: Electronic Supplementary Material (ESM) is supplied by the authors for the benefit of readers. This feature is intended to provide additional information, context, and/or dimension to complement articles. Readers should be aware that unlike the main print manuscript, ESM may not undergo extensive peer review and typically is posted as supplied by the authors without editing, proofreading, accuracy checking, copyediting, or typesetting; it is published online only.

TABLE OF CONTENTS

Table S1 Sensitivity analysis, impact of coughing variables on model performance

Table S2 Sensitivity analysis, impact of dysphonia variables on model performance

Table S3 Sensitivity analysis, impact of stridor on model performance

Table S4 Sensitivity analysis, comparison of the initial model A with the simplified model A (SARI with clinical factors) after sensitivity analysis

Table S1. Sensitivity analysis, impact of coughing variables (dry cough, productive cough, impaired expectoration) on model performance, comparison between multivariable mixed effects logistic regression models in the entire study cohort (*N* = 374).

**Characteristics**	SARI with clinical factors<br>OR (95% CI)	Without productive cough and impaired expectoration<br>OR (95% CI)	Without dry cough and impaired expectoration<br>OR (95% CI)	Without dry and productive cough<br>OR (95% CI)
**SARI** [0-12]	1.35 (1.19 to 1.53)	1.37 (1.20 to 1.56)	1.36 (1.22 to 1.51)	1.38 (1.21 to 1.57)
**Clinical factors**				
Age [years]	1.01 (0.99 to 1.03)	1.01 (0.99 to 1.03)	1.01 (0.99 to 1.03)	1.01 (0.99 to 1.03)
Height [cm]	1.01 (0.98 to 1.04)	1.01 (0.98 to 1.04)	1.01 (0.99 to 1.04)	1.01 (0.98 to 1.04)
Dysphagia	2.31 (1.25 to 4.27)	2.72 (1.43 to 5.17)	2.11 (1.20 to 3.70)	2.41 (1.29 to 4.49)
Weak voice or phonation difficulties	1.65 (0.70 to 3.90)	1.83 (0.75 to 4.48)	1.49 (0.68 to 3.26)	1.59 (0.67 to 3.80)
Whispering or aphonia	2.68 (0.80 to 8.92)	3.22 (0.92 to 11.28)	2.34 (0.77 to 7.07)	2.70 (0.79 to 9.17)
Dry cough	0.51 (0.22 to 1.14)	0.51 (0.23 to 1.14)	-	-
Productive cough	1.33 (0.60 to 2.92)	-	1.18 (0.61 to 2.28)	-
Impaired expectoration	1.23 (0.48 to 3.12)	-	-	1.14 (0.48 to 2.71)
Stridor	0.83 (0.13 to 5.25)	0.84 (0.13 to 5.51)	0.82 (0.14 to 4.67)	0.79 (0.12 to 5.27)
ICC	0.20	0.24	0.19	0.23
AICc	479.2	475.8	478.8	478.5

SARI: simplified airway risk index; ICC: intra-class correlation; AICc: Akaike information criterion with correction for small sample size; data are presented as odds ratio (OR) with 95% confidence interval (95% CI)

Table S2. Sensitivity analysis, impact of dysphonia variables (weak voice or phonation difficulties, whispering or aphonia) on model performance, comparison between multivariable mixed effects logistic regression models in the entire study cohort (*N* = 374).

**Characteristics**	SARI with clinical factors<br>OR (95% CI)	Without whispering or aphonia<br>OR (95% CI)	Without weak voice or phonation difficulties<br>OR (95% CI)
**SARI** [0-12]	1.35 (1.19 to 1.53)	1.36 (1.19 to 1.55)	1.34 (1.21 to 1.50)
**Clinical factors**			
Age [years]	1.01 (0.99 to 1.03)	1.01 (0.99 to 1.03)	1.01 (0.99 to 1.03)
Height [cm]	1.01 (0.98 to 1.04)	1.01 (0.98 to 1.04)	1.01 (0.99 to 1.04)
Dysphagia	2.31 (1.25 to 4.27)	2.47 (1.29 to 4.76)	2.38 (1.35 to 4.20)
Weak voice or phonation difficulties	1.65 (0.70 to 3.90)	2.63 (1.15 to 6.00)	-
Whispering or aphonia	2.68 (0.80 to 8.92)	-	3.57 (1.30 to 9.75)
Dry cough	0.51 (0.22 to 1.14)	0.48 (0.20 to 1.13)	0.53 (0.25 to 1.14)
Productive cough	1.33 (0.60 to 2.92)	1.44 (0.63 to 3.30)	1.30 (0.62 to 2.72)
Impaired expectoration	1.23 (0.48 to 3.12)	1.34 (0.50 to 3.57)	1.32 (0.55 to 3.19)
Stridor	0.83 (0.13 to 5.25)	0.91 (0.13 to 6.26)	0.79 (0.14 to 4.57)
ICC	0.20	0.25	0.20
AICc	479.2	479.9	478.8

SARI: simplified airway risk index; ICC: intra-class correlation; AICc: Akaike information criterion with correction for small sample size; data are presented as odds ratio (OR) with 95% confidence interval (95% CI)

Table S3. Sensitivity analysis, impact of stridor on model performance, comparison between multivariable mixed effects logistic regression models in the entire study cohort ($N = 374$).

**Characteristics**	SARI with clinical factors<br>OR (95% CI)	Without stridor<br>OR (95% CI)
**SARI** [0-12]	1.35 (1.19 to 1.53)	1.35 (1.21 to 1.50)
**Clinical factors**		
Age [years]	1.01 (0.99 to 1.03)	1.01 (0.99 to 1.03)
Height [cm]	1.01 (0.98 to 1.04)	1.01 (0.98 to 1.04)
Dysphagia	2.31 (1.25 to 4.27)	2.23 (1.26 to 3.97)
Weak voice or phonation difficulties	1.65 (0.70 to 3.90)	1.60 (0.71 to 3.61)
Whispering or aphonia	2.68 (0.80 to 8.92)	2.56 (0.82 to 7.99)
Dry cough	0.51 (0.22 to 1.14)	0.51 (0.24 to 1.09)
Productive cough	1.33 (0.60 to 2.92)	1.30 (0.62 to 2.73)
Impaired expectoration	1.23 (0.48 to 3.12)	1.22 (0.51 to 2.96)
Stridor	0.83 (0.13 to 5.25)	-
ICC	0.20	0.19
AICc	479.2	477.3

SARI: simplified airway risk index; ICC: intra-class correlation; AICc: Akaike information criterion with correction for small sample size; data are presented as odds ratio (OR) with 95% confidence interval (95% CI)

Table S4. Sensitivity analysis, comparison of the initial model A with the simplified model A (SARI with clinical factors) after sensitivity analysis in the entire study cohort ($N = 374$).

**Characteristics**	SARI with clinical factors<br>OR (95% CI)	SARI with simplified clinical factors (without productive cough, impaired expectoration, weak voice or phonation difficulties and stridor)<br>OR (95% CI)
**SARI** [0-12]	1.35 (1.19 to 1.53)	1.34 (1.34 to 1.35)
**Clinical factors**		
Age [years]	1.01 (0.99 to 1.03)	1.01 (1.00 to 1.01)
Height [cm]	1.01 (0.98 to 1.04)	1.01 (0.98 to 1.04)
Dysphagia	2.31 (1.25 to 4.27)	2.58 (2.57 to 2.59)
Weak voice or phonation difficulties	1.65 (0.70 to 3.90)	-
Whispering or aphonia	2.68 (0.80 to 8.92)	3.81 (1.40 to 10.41)
Dry cough	0.51 (0.22 to 1.14)	0.61 (0.30 to 1.23)
Productive cough	1.33 (0.60 to 2.92)	-
Impaired expectoration	1.23 (0.48 to 3.12)	-
Stridor	0.83 (0.13 to 5.25)	-
ICC	0.20	0.19
AICc	479.2	473.8

SARI: simplified airway risk index; ICC: intra-class correlation; AICc: Akaike information criterion with correction for small sample size; data are presented as odds ratio (OR) with 95% confidence interval (95% CI)
